# Prevalence and factors associated with opioid use disorder among adolescents with sickle cell disease in Mulago hospital, Uganda

**DOI:** 10.1186/s13034-024-00790-4

**Published:** 2024-08-01

**Authors:** Claire Kwagala, Deogratias Munube, Catherine Abbo, Wilson Winstons Muhwezi, Emmanuel Kiiza Mwesiga

**Affiliations:** 1 Department of Psychiatry, School of Medicine, College of Health Sciences, Makerere University, P. O. Box 7062, Kampala, Uganda; 2 Department of Paediatrics and Child Health, School of Medicine, College of Health Sciences, Makerere University, Kampala, Uganda

**Keywords:** Sickle cell disease, Opioid use disorder, Depression, Anxiety, Low resource setting, Uganda

## Abstract

**Background:**

Opioid use disorder (OUD) among adolescents with sickle cell disease (SCD) patients increases their risk of complications from sickle cell disease, such as infections, stroke, acute chest syndrome, sudden death, and organ failure. This negatively impacts families, communities, the national health system, and the economy. This study aimed to determine the prevalence and factors associated with opioid use disorder among adolescents with SCD at Mulago Hospital Uganda.

**Methods:**

This study was carried out at the Sickle Cell Clinic of Mulago Hospital, the national referral hospital in Uganda. The study participants were adolescents aged 10 to 19 years. Following informed consent/ assent, a sociodemographic questionnaire, the WHO Alcohol, Smoking and Substance Involvement Screening Test - Young (ASSIST-Y), the Beck Depression Inventory-II (BDI II), and Generalized Anxiety Disorder − 7 (GAD-7) questionnaires were used to collect data. Data was entered in EpiInfo and analyzed in STATA 15.

**Results:**

The prevalence of opioid use disorder was 5.3%. The significant risk factor was increasing depressive score AOR: 1.11(95% CI: 1.01–1.22, *p* = 0.035), while living with a family was protective against opioid use disorders AOR: 0.01; (95% CI: 0.0004, 0.27, *p* = 0.007).

**Conclusion:**

There was a significant problem of OUD among adolescents with SCD. There is, therefore, needed to integrate screening of OUD and mental illnesses like depression among adolescents with SCD and to emphasize the importance of family support in their care.

## Background

The main clinical feature of Sickle Cell Disease (SCD) is acute painful crises, which include Vaso-Occlusive pain and splenic sequestration, among others that are often chronic [1]. Chronic SCD pain for adolescents aged 10–18 has been associated with poorer psychosocial outcomes placing them at risk of depression and anxiety [2]. Moreover, the painful crises result in patients’ reliance on opioid medication like morphine used in Uganda, placing the population at a greater risk of Opioid Use Disorder (OUD). Opioid use disorder (OUD), also known as opioid misuse, opioid dependence or opioid abuse, is a type of substance use disorder [3]. Opioid analgesic drugs, when misused, are potentially toxic, with a common spectrum of adverse events [4]. Inappropriate dosing can result in fatalities, particularly related to respiratory depression and excessive sedation [4]. The management of pain among patients with sickle cell disease (SCD) depends mostly on the use of opioids [5, 6]. The chronic nature of SCD and, thus, continuous use of opioids leads to physiological dependence and tolerance, where they require increased opioid doses to achieve analgesia [7].

Prevalence of OUD in adolescents with SCD has been observed in developing nations such as Nigeria, where patients have developed a dependence on opioids like Pentazocine [8]. In this study, twenty-four per cent (24%) of the studied population injected Pentazocine, and 17.6% were dependent on the opioid as per the ICD-10 criteria for dependence [8]. This is also supported by other studies on the effect of long-term prescription drug use being a contributing factor in SUD with adults developing OUD due to prolonged exposure to opioids as analgesics. In the US, opioid use among the SCD population is reported at 10% [9]. Adolescents, however, are at a higher risk of OUD due to enhanced brain neuroplasticity and an underdeveloped frontal cortex that is crucial in self-control and regulation [10].

Risk factors for opioid use disorder among SCD patients include personal or family history of substance use, substance use by the peer group, past or current mental illness diagnosis of depression and anxiety, history of sexual abuse, frequent painful crises, avascular necrosis, higher pain intensity and higher hospital and emergency department use [11–16]. However, existing literature on prevalence and factors associated with OUD among the SCD adolescent population is scanty as the majority of the studies focus on adults with SCD [11, 16–18], despite adolescence being crucial years in forming habits that persist into adulthood [19]. Consequently, there remains unidentified OUD in the adolescent SCD population, posing various neurological, behavioural, and medical complications for patients. Unidentified OUD increases the likelihood of individuals engaging in substance abuse, committing crimes, and transmitting infections [12, 19, 20]. The impact also spills into communities, emphasizing the need to comprehend the phenomenon. Research on the prevalence of OUD in the SCD population is vital in improving screening, interventions and treatments that consider the risk factors that are unique to this population. This study aimed to fill this gap by investigating the prevalence and factors associated with OUD among adolescents with SCD in Mulago Hospital, Uganda.

## Methods

### Study design and setting

This cross-sectional study aimed to determine the prevalence of opioid use disorder among adolescents with sickle cell disease (SCD) at Mulago Hospital, Uganda, and to explore the associated factors. This study was conducted at the Sickle Cell Clinic (SCC) in the country’s national referral and teaching hospital. The SCC receives approximately 7000 patients, with 3800 (54%) being adolescents. The standard of care includes quarterly reviews, although patients can also be seen on a need-based basis. They are on routine medications, such as folic acid, Penicillin V, Hydroxyurea, and vaccinated against pneumococcal infections. Routine laboratory tests include a complete blood count and renal function tests. The standard opioid used at the Mulago Hospital SCC in Uganda is morphine. Tramadol is another opioid used to treat painful crises in SCD and it is accessible over the counter.

### Study population and sample size

Adolescents aged 10–19 years with SCD and undergoing routine sickle cell care at Mulago Hospital were recruited to participate in the study. Adolescents were eligible to participate if (i) they were aware of their SCD diagnosis, (ii) they gave consent (> 18 years) or assent (< 18 years), and (ii) the caregiver consented (< 18 years). Adolescents were excluded if they could not sustain the interview because of pain or unstable vitals.

The required sample size was determined using the Leslie Kish formula [21], with a power of 80%, confidence level of 95%, and precision of 5% between the true prevalence of opioid use disorder and the estimated prevalence from the sample. This calculation resulted in a minimum sample size of 384 patients.

### Study procedure

The SCC clinic is open Monday to Friday and receives approximately 30 adolescent patients daily. Trained research assistants worked together with the staff at Mulago Hospital SCC. On a given day, primary care providers approached potential adolescent participants with their caregivers as they waited to be attended to at the clinic. We invited every third adolescent to participate in the study using systematic random sampling, making ten potential participants available daily. The research assistants ensured that the adolescents had been attended to by the clinic staff and had received their treatment before being recruited for the study. They briefly introduced the study and assessed their eligibility before referring them to the research team. A more detailed description of the study was provided by the research assistants, who provided written informed consent. Caregivers gave consent for adolescents aged < 18 years, and the adolescents assented to participate in the study. Study subjects were consecutively enrolled, with an average of five participants recruited daily, resulting in a final sample size of 381 (out of the 384 proposed) participants recruited over 16 weeks, from July to September 2021.

Only those participants who provided consent were administered a set of questionnaires. Once consent was obtained, participants were taken to a study room to ensure confidentiality and to ensure that we did not interfere with the daily flow of work at the Sickle Cell Clinic, and then the study instruments were administered. It took approximately 30 min to complete the set of questionnaires. Participants with opioid use disorders were referred to an adolescent mental health clinic for adequate treatment.

### Data collection instruments

A researcher-designed demographic questionnaire collected information on adolescents’ age, sex, religion, tribe, education level, employment status, and living situation. Clinical variables, including the frequency of pain crises, were obtained from participants. The duration of opioid therapy and concurrent opioid use were obtained from patients’ files.

Opioid use disorder was assessed using the Alcohol, Smoking, and Substance Involvement Screening Test - Youth (ASSIST-Y) [22]. The ASSIST-Y is used to assess substance use and related problems among adolescents. The tool consists of 8 questions focusing on the frequency, quantity, and impact of substance use. Each question on the ASSIST-Y is answered using a Likert scale, ranging from ‘never’ to ‘almost daily.’ Higher scores reflect more frequent or problematic substance use. The responses are summed to generate a total score for each substance assessed.

In our study, we used the following cut-off points to categorize opioid use disorder: Low Risk: A total score of 0–2, indicating minimal substance use with little to no risk of OUD. Moderate Risk: A total score of 3–6, suggesting moderate substance use with potential signs of OUD. High Risk: A total score of 7 or higher, reflecting significant substance use and a high likelihood of having OUD. Participants with scores in the high-risk category are classified as having opioid use disorder (OUD), while those in the low and moderate risk categories are considered not to have OUD.

Studies on the original ASSIST show good psychometric properties for the alcohol subscale, particularly among adults, with sensitivities ranging from 0.67 to 0.83 and specificities between 0.60 and 0.79. Few studies have reported the psychometric properties of the ASSIST-Y [23].

The Beck’s Depression Inventory Second Edition (BDI-II) is a 21–item multiple-choice self-report instrument intended to assess the existence and severity of symptoms of depression. Each of the 21 items corresponding to a symptom of depression was summed up to obtain a single score. Each item is rated on a four-point scale ranging from 0 to 3. A total score of 0–13 was considered the normal range; 14–19 is mild, 20–28 is moderate, and 29–63 is severe. The BDI-II has demonstrated high reliability, regardless of its population (Medical University of South Carolina). It has an internal consistency of 0.9, and its retest reliability ranges from 0.73 to 0.96. A high coefficient alpha (0.93) and construct validity have been established, and it can differentiate depressed from non-depressed patients [24].

The Generalized Anxiety Disorder Scale (GAD-7) was used to assess the generalized anxiety symptoms. The GAD-7 is a 7-item scale that investigates how often the patient has been bothered by seven different symptoms of anxiety during the last two weeks with response options such as: “not at all,” “several days”, “more than half the days,” and “nearly daily” scored as 0, 1, 2, and 3, respectively [25]. Scores of 5, 10, and 15 were used as cut-off points for mild, moderate, and severe anxiety, respectively. If we use a threshold of 10, the GAD-7 assumes an excellent sensitivity of 89% and specificity of 82% for generalized anxiety disorder (26). GAD-7 scale was found to have an internal consistency of 0.92 (Cronbach α = 0.92) as well as a good test-retest reliability of 0.83 (intraclass correlation = 0.83) [26].

### Data management and quality control

The study’s internal validity was ensured through the interviewers’ adequate training. A linguistic expert translated the questionnaires and tools from English into the Luganda language. The questionnaires were corrected for errors before data entry. The supervisors closely checked all the stages of the study. A statistician was consulted throughout the study, including during the proposal development.

All data were confidentially obtained with strict observance and respect for patient privacy by using unique study identification numbers instead of their names. The Principal Investigator (PI) examined each completed questionnaire for completeness. Trained data clerks using the EpiInfo computer software and created an electronic database from the raw data. A double entry was conducted to minimize entry errors. A database was created for safe data storage. All collected data were backed up on a hard drive and stored under lock and key at the study site. The data on the computer were secured with a password known only to the PI.

### Data analysis

Data from the tools and questionnaires were coded and entered using the EpiInfo package, then exported to the STATA 15 package for analysis. Descriptive statistics were used to describe the participants’ sociodemographic characteristics, which are presented as frequencies and percentages.

For continuous variables, means and standard deviations (mean ± SD) were used for normally distributed data, while medians and interquartile ranges (IQR) were used for non-normally distributed data.

The primary dependent variable was opioid use disorder (OUD), which is a binary outcome expressed as percentages (cases with OUD and non-cases without OUD). Other variables, including depression and anxiety, were independent predictors of OUD.

Chi-square tests were used to assess the relationship between demographic factors and OUD. A series of logistic regression models were used to evaluate the association between various factors and OUD which included; (1) Crude Analysis: Each factor’s association with OUD was assessed individually. (2) Fully Adjusted Penalized Logistic Model: This model was employed to assess the independent association of risk factors with OUD, accommodating very sparse outcome data for some variables, such as living status, which had very few counts for patients with OUD. 3.Backward Elimination Method: This method was used at a 0.05 level of significance to control for overfitting and improve the generalizability of the study results.

The magnitude of association was reported using odds ratios (ORs) with their respective 95% confidence intervals (CIs) and a p-value of < 0.05 was considered statistically significant. For both the bivariate and regression analyses, we restricted our analysis to participants (*N* = 235) who had used opioids in the past 3 months. Specifically, we assessed opioid use disorder with the question, “In the past 3 months, how often have you used the substance selected?” This question was converted to a binary variable for analysis.

### Ethics and approvals

Approval for the study was obtained from the Department of Psychiatry and School of Medicine Research Ethics Committee (Mak-SOMREC-2021-72) at Makerere University. Administrative clearance was also secured from the Mulago Hospital and the Clinical Head of the Sickle Cell Clinic. Written informed consent was obtained from individuals older than 18 and assent from those under 18. For participants aged < 18, consent was obtained from their parents or legal guardians. Consent and assent documents were translated into Luganda, the participants’ primary language. When subjects could not read, the Principal Investigator (PI) or a research assistant read the document aloud. All consenting individuals signed the relevant forms; those unable to write provided consent by fingerprint, witnessed by the PI or their representative. Copies of signed or fingerprinted consent and assent forms were provided to potential study participants. Those who declined consent or assent were not prejudiced, and their routine healthcare continued uninterrupted. To ensure confidentiality, identification numbers, rather than participant names, were used, and all data were securely stored under lock and key, including passwords for computer-stored data.

## Results

This study included 381 adolescents with SCD, with a median age of 13 years (IQR: 11, 15). At least half of the participants were male (50.1%). The majority of the adolescents were Christians (63%) and single (97%, *n* = 369), with a primary education level (65%, *n* = 249) and living with their families (91%, *n* = 347). The mean number of painful crises in the past year was 2 (SD = 1.9). One-third of the study participants (32%, *n* = 120) were found to have mild general anxiety disorder, while those with severe anxiety (1%, *n* = 4) and (5.2%, *n* = 18) had severe depression. See Table 1.

**Table 1:** Background and clinical characteristics of study participants (*N* = 381)

Variable	Frequency	Percentage
**Age** 10–14 15–19 Median (IQR) 13 (11,15)	283 98	74.3 25.7
**Sex** Male Female	191 190	50.1 49.9
**Religion** Christian Islam	240 141	63.0 37.0
**Marital status** Single Married	369 12	96.9 3.1
**Education level** Primary Secondary and above	249 132	65.4 34.6
**Living situation** Lives alone Living with family	34 347	8.9 91.1
**Employment status** Unemployed/In school Employed	360 21	94.5 21
**Number of painful crises past year** None Two and more ***Mean (SD)*** 2 (1.9)	126 255	33.1 66.9
**General anxiety disorder** No notable anxiety Mild Moderate Severe *(min/max score: 0–16)*	206 120 51 4	54.1 31.5 13.4 1.0
**Depression** Minimal Mild Moderate Severe *(min/max score: 1–40)*	293 16 21 18	84.2 4.6 6.0 5.2

The overall prevalence of opioid use disorder among adolescents with SCD was 5.3% (20/381). The prevalence of 3-month opioid use disorder among adolescents with SCD was 8.5% (20/235). The bivariate analysis was performed using the 3-month opioid use disorder as the primary outcome.

The average age of patients with opioid use disorder was 16 years, compared to 13 years for those without the disorder, indicating that adolescents with OUD were significantly older. 90% of participants with opioid use disorder were male, and 10% were female, suggesting a significant association between male gender and OUD.

Regarding religious affiliation, 60% of participants with opioid use disorder were Christians, while 40% were Muslims. Furthermore, the analysis revealed that age, sex, marital status, highest level of education, living situation, employment status, and depression were significantly associated with opioid use disorder.(Table 2).

**Table 2:** Bivariate analysis for socio-demographics by opioid use disorder

Variable	Opioid use disorder			
	Yes (*n* = 20) %	No (*n* = 215) %	Total (*N* = 235) %	Chi-square or t-test values
Age in years *[****mean***,*** SD****]*	16.4 (1.54)	13.48 (2.39)	13.73 (2.47)	-5.32***
**Sex**				17.07***
Male	(18) 90.00	(90) 41.86	(108) 45.96	
Female	(2) 10.00	(125) 58.14	(127) 54.04	
**Religion**				0.06
Christian	(12) 60.00	(135) 62.79	(147) 62.55	
Islam	(8) 40.00	(80) 37.21	(88) 37.45	
**Marital status**				4.41*
Married	(3) 15.00	(9) 4.19	(12) 5.11	
Single	(17) 85.00	(206) 95.81	(223) 94.89	
**Education**				9.41**
Primary	(5) 25.00	(130) 60.47	(135) 57.45	
Secondary or tertiary	(15) 75.00	(85) 39.53	(100) 42.55	
**Level of pain**				0.001
Yes	(13) 65.00	(139) 64.65	(152) 64.68	
No	(7) 35.00	(76) 35.35	(83) 35.32	
**Living situation**				122.06***
Lives alone	(16) 80.00	(7) 3.26	(23) 9.79	
Lives within a family	(4) 20.00	(208) 96.74	(212) 90.21	
**Employment**				54.93***
Unemployed	(12) 60.00	(211) 98.14	(223) 94.89	
Employed	(8) 40.00	(4) 1.86	(12) 5.11	
Anxiety *[****mean***,*** SD****]*	4.75 (4.48)	4.01 (3.86)	4.07 (3.91)	-0.80
Depression *[****mean***,*** SD****]*	21.15 (11.04)	6.50 (9.12)	7.75 (10.14)	-6.73***

*p<0.05; **p<0.01; ***p<0.001

Unadjusted results indicated that age, sex, religion, education, living situation, employment, and depression were significantly associated with opioid use disorder among adolescents with sickle cell disease.

Thank you for pointing out the error in the interpretation of odds ratios. Here is the revised text with the correct interpretations:

Specifically, the crude odds of older age, being female, level of education, living alone, being employed, and having depression increased the odds of having OUD (see Table 3). The adjusted results in model 1 showed that living situations and depression were the only risk factors significantly associated with opioid use disorder among sickle cell patients. Specifically, the odds of getting OUD among those living within a family were 0.01 times lower than those living alone (aOR = 0.01, 95% CI: 0.0004, 0.27, *p* < 0.05). The odds of having OUD increased by 1.11 times for each additional mean score of depression (aOR = 1.11, 95% CI: 1.01, 1.22, *p* < 0.05).

In model 2, the results showed that sex (being female) (aOR = 0.11, 95% CI: 0.01, 0.64, *p* < 0.05), living situation (living with family) (aOR = 0.02, 95% CI: 0.04, 0.08, *p* < 0.05), and depression (aOR = 1.10, 95% CI: 0.04, 0.08, *p* < 0.05) were significantly associated with opioid use disorder among adolescents with sickle cell disease.

**Table 3:** Factors associated with opioid use disorder as observed in the crude and adjusted analyses (adjusted for age, sex, and educational level)

	Crude OR, 95%CI	*p*-value	Model 1: fully adjusted OR, 95%CI	*p*-value	Model 2: backward adjusted OR, 95%CI	*p*-value
Age in years	**1.61 (1.31**,** 1.98)**	**0.001**	1.11(0.68, 1.80)	0.667		
**Sex (ref: male)**						
Female	**0.08 (0.02**,** 0.35)**	**0.001**	0.17(0.02, 1.16)	0.071	**0.11(0.01**,** 0.64)**	**0.015**
**Religion (ref: Christian)**						
Islam	1.13 (0.44, 2.87)	0.805	1.44(0.31, 6.76)	0.640		
**Marital status (ref: single)**						
Married	4.03 (0.99, 16.33)	0.050	0.41(0.01, 30.36)	0.686		
**Education (ref: primary)**						
Secondary or tertiary	**2.47 (1.16**,** 5.29)**	**0.019**	0.30(0.02, 5.34)	0.417		
**Level of pain (ref: No)**						
Yes	1.02 (0.38, 2.65)	0.975	14.02(0.76, 259.27)	0.076		
**Living situation (ref: lives alone)**						
Lives within a family	**0.01 (0.002**,** 0.03)**	**0.001**	**0.01(0.0004**,** 0.27)**	**0.007**	**0.02(0.04**,** 0.08)**	**< 0.001**
**Employment (ref: employed)**						
Unemployed	**0.03 (0.01**,** 0.11)**	**0.001**	0.72(0.05, 9.67)	0.315		
**General anxiety disorder**	1.05 (0.93, 1.18)	0.421	1.13(0.89, 1.42)	0.248		
**Depression**	**1.12 (1.07**,** 1.16)**	**0.001**	**1.11(1.01**,** 1.22)**	**0.035**	**1.10(1.03**,** 1.17)**	**0.005**

## Discussion

This study presents the prevalence and factors associated with OUD among adolescents with SCD in Mulago Hospital, Uganda. We found a prevalence of 5.3% of OUD, with living situations and depression being the main factors contributing to OUD among the adolescents living with SCD. The prevalence highlights that the SCD population is not considered at risk of OUD due to the reported low prevalences in the few studies that have studied the population [5, 9]. Despite the paucity of evidence, especially in Sub-Saharan Africa (SSA), the growing concern about OUD prevalence in the general population makes our study relevant, as chronic conditions like SCD increase the likelihood of OUD [27]. Other studies looking at adult populations also report higher dependence prevalence where, for instance, Benin had 17.6% [8], which was lower than what was reported in the USA for non-cancer-related pain at 41.3% [28]. Substance Use Disorder (SUD) in adults with SCD in Nigeria was reported to be at 5%, with the majority reporting opioid use [29] and Pentazocine, which has a short half-life, thus predisposing the patient to addiction, has been specifically reported on in adult and pediatric populations [8, 16, 30, 31]. Again, despite the relatively lower prevalence reported in our study, it is important to note that it indicates that the adolescent SCD population is likely to present with OUD, increasing the disease burden. Therefore, medical professionals need to screen adolescents with SCD keenly for OUDs in their practice to identify problems and offer evidence-based care for management.

Thank you for your feedback. We have revised the sentence for clarity. Here is the updated text;

Our study highlights factors such as one’s living situation and untreated psychological problems as affecting OUD among adolescents with SCD. Specifically, we report a lower likelihood of OUD if an adolescent is living with family as opposed to living alone, which is consistent with findings from the US. In the US study, high levels of parental warmth and reduced parental supervision were positively correlated with lower rates of opioid misuse among 12 to 14-year-olds. (t=-2.42, b=-0.112, *p* = 0.19) [32]. Thus, having an available social support system for the population identified at risk of developing OUDs due to chronic conditions such as SCD is recommended.

Depression was also associated with higher OUD among our focus population, which is similarly reported in a systematic review looking at the role of anxiety and depression on OUD by Rogers and colleagues [33]. The findings point to a link between untreated psychological problems, specifically depression, and increased pain in chronic conditions [26]. Chronically ill patients with depression report more physiological symptoms [34].

Among patients with SCD, reports indicate that pain intensity is often higher and more frequent, with a greater impact on their lives. The literature consistently highlights a link between increased pain and depression. [35–49]. Low internalized stigma was associated with depressive symptoms and pain [40], while discrimination in healthcare settings was linked to increased clinical pain severity, stress, depression, and sleep problems [50]. Furthermore, depression was associated with reduced health-related Quality of Life scores (HRQoL) among adults with SCD in the US [ [35]. This warrants the need to screen for potential psychosocial determinants of OUD before the initiation of opioid therapy for pain management among adolescents with SCD.

Our study was limited in its evaluation of other factors that would put the adolescent SCD population at risk of OUD. This includes the inability of the various health systems to effectively control the pain crises, disease-associated chronic pain due to osteomyelitis, necrosis, fractures, readily accessible to uncontrolled opioids, genetic predisposition, and a lack of knowledge of associated side effects [24, 30]. The study also missed out on an opportunity to study the HealthCare Workers’ (HCWs) knowledge and attitude about OUD in the population to highlight the need for a multidisciplinary approach to the care of adolescents living with SCD given their unique challenges as the HCWs have been reported to overestimate opioid use and misuse among SCD patients [51–53]. Determining the type of opioid in use among the Ugandan population would have also built onto the scarce knowledge of the specific type, method, and dosage administered [54].

### Strengths and limitations of the study

To the best of our knowledge, this is among the first studies to highlight the prevalence and factors associated with opioid use disorder among adolescents in our African settings that look at various opioids. Therefore, this study gives insight into opioid use disorder from the African perspective and also adds to the knowledge gap in this area by providing baseline information for opioid use disorder among adolescents with Sickle Cell disease.

This study did not assess the effect of some important variables, such as history of other substance abuse and the effect of urban/rural differences, which have been shown to have an association with opioid use disorder in other studies [19, 21, 55]. Furthermore, other variables such as stigma could not be accounted as we did not collect the data. Recall bias caused by differences in the accuracy or completeness of the recollections retrieved by study participants regarding events, times or experiences from the past was a methodological limitation due to the use of interviews or questionnaires. In addition, social desirability bias was another challenge in this study, as the respondents may tend to answer questions in a manner that others will view favourably. In this study, these adolescents are likely to under-report opioid use, as it may be perceived as a bad” or undesirable behavior.

## Conclusions

Given the potential for long-term and detrimental consequences of prescription opioid use among adolescents in our community, it is imperative to identify the burden and determinants of opioid use disorder among those who are usually in contact with prescription drugs for pain management, such as those with SCD as highlighted in this study. In this study, we found a prevalence of 5.3% of opioid use disorder among adolescents with SCD, with family and depression associated with OUD. The burden and determinants of opioid use disorder among adolescents with SCD, as highlighted in this study, will facilitate the roadmap for prevention and development of intervention efforts aimed at reducing the consequences of opioid use disorder among adolescents with SCD. Continuous screening of adolescents with SCD on opioid therapy for OUD is essential. It can be achieved by training the staff at the Sickle Cell Clinic in Mulago and all health care providers for adolescents with SCD on how to identify adolescents with probable OUD. Incorporation of screening tools like ASSIST in the assessment of adolescents with SCD on opioid therapy would combat the substantial prevalence of OUD. This would offer holistic care and a better prognosis for SCD.

## Data Availability

No datasets were generated or analysed during the current study.

## Abbreviations

OUD: Opioid Use Disorder
SCD: Sickle Cell Disease
SUD: Substance Use Disorder
HRQoL: Health-related Quality of Life scores
